# Epigenetic insights into cancer metastasis via lineage trans-differentiation

**DOI:** 10.1016/j.gendis.2026.102282

**Published:** 2026-06-06

**Authors:** Cong Zou, Caixia Xia, Hongwei Peng, Shenjuan Li, Jia Shang, Wan Xiang, Haoli Ma, Fangjin Chen, Xinyue Cao, Gang Wang

**Affiliations:** a Department of Biological Repositories, Zhongnan Hospital of Wuhan University, Wuhan, Hubei 430071, China; b Human Genetic Resources Preservation Center of Hubei Province, Wuhan University, Wuhan, Hubei 430071, China; c Dean Office, Zhongnan Hospital of Wuhan University, Wuhan, Hubei 430071, China; d Laboratory of Precision Medicine, Zhongnan Hospital of Wuhan University, Wuhan, Hubei 430071, China; e Brain Glioma Center & Department of Neurosurgery, Zhongnan Hospital of Wuhan University, Wuhan, Hubei 430071, China; f High Performance Computing Center, The Peking-Tsinghua College of Life Sciences, Peking University, Beijing 100091, China

The molecular etiology of cancer metastasis, which is responsible for more than 90% of cancer-related mortality, has long been obscured by a fundamental paradox: metastatic lesions share nearly identical driver mutation landscapes with their primary tumor counterparts, but exhibit profoundly divergent transcriptional and phenotypic profiles.^1^ This genomic-phenotypic dissociation strongly implicates nongenetic, epigenetically mediated mechanisms as principal determinants of metastatic competence. The cancer epigenome, long recognized as a domain of therapeutic opportunity,^2^ has until recently lacked a coherent mechanistic framework connecting its dysregulation to the generation of metastasis-competent clones (see Fig. 1).

**Figure 1:**
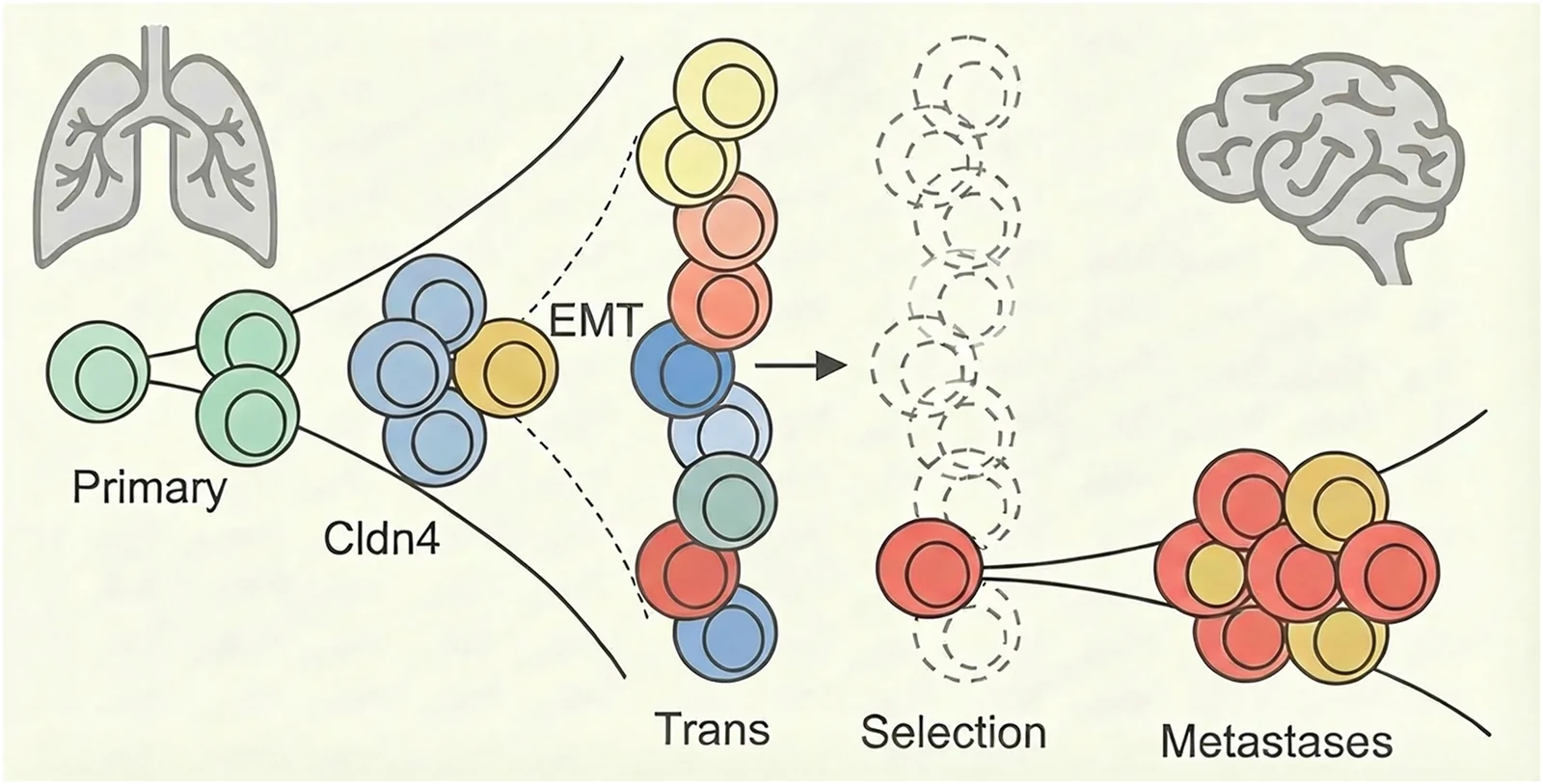
Lineage trans-differentiation and selective bottleneck during metastatic progression. Schematic model of metastasis evolution from a lung primary tumor. Primary tumor cells transition into a Cldn4^+^ high-plasticity state, followed by epithelial–mesenchymal transition (EMT) and subsequent lineage trans-differentiation (“Trans”) into alternative-lineage programs (colored cell states). Metastatic dissemination is constrained by a stringent selection bottleneck (dashed outlines), such that only a small subset of trans-differentiated cells successfully seeds and expands to form overt metastases at distant sites (illustrated by the brain icon). The curved trajectories indicate progressive state changes; the branching lines denote clonal diversification and outgrowth.

In a recent study published in *Research*, Xiao et al^3^ systematically addressed this outstanding question, demonstrating that lineage trans-differentiation, a process whereby cancer cells progressively abandon their original cellular identity and acquire transcriptional programs characteristic of developmentally distant lineages, is a universal hallmark of cancer metastasis, driven by epigenomic reprogramming and mechanistically linked to oncogenic mitogen-activated protein kinase (MAPK) signaling.

By integrating multiple single-cell genomic datasets from a genetically defined *Kras*^CA^; *Trp53*^KO^ mouse lung adenocarcinoma model with publicly available human tumor data, Xiao et al reconstructed the evolutionary trajectory from normal alveolar type 2 cells to distant metastases at single-cell resolution. By employing an orthogonal combination of pseudotime inference, genetic lineage tracing, and single-cell replicational age estimation via the EpiTrace algorithm,^4^ the authors define a step-wise metastatic evolutionary axis: alveolar type 2 cells → bronchioalveolar stem cells → primary cancer → claudin 4-positive (Cldn4^+^) high-plasticity state → epithelial–mesenchymal transition → metastases. Three key epigenomic transitions punctuate this trajectory. First, during pre-metastatic evolution, cancer cells undergo progressive dedifferentiation, in which lineage-specific transcription factors are down-regulated (including NKX2-1, CEBPA, FOXA2), and a fetal/stem-like intermediate state is adopted. Second, at the epithelial–mesenchymal transition stage, a global increase in chromatin accessibility enables alternative-lineage transcription factors, previously expressed but epigenomically constrained, to engage their target loci and activate ectopic gene programs. Third, the resulting trans-differentiated metastatic cells express transcriptional and epigenomic signatures characteristic of neuroglial, endothelial, and stromal lineages, which are entirely distinct from their epithelial cells of origin. Crucially, population bottleneck analysis revealed that only approximately 5%–8% of pre-metastatic Cldn4^+^ cells successfully traverse the epithelial–mesenchymal transition and complete this trans-differentiation process, providing a quantitative explanation for the clinical rarity of metastatic dissemination despite the widespread primary tumor burden.

The concept of lineage trans-differentiation as a metastatic mechanism resolves several longstanding inconsistencies in cancer biology. The prevailing epithelial–mesenchymal transition hypothesis, while delineating critical intermediate states, has struggled to account for the observation that fully epithelial–mesenchymal transitioned cells tend toward quiescence rather than proliferative metastatic outgrowth. Similarly, the fetal-like transcriptional state is characterized by intermediate rather than endpoint metastatic phenotypes. By demonstrating that fetal-like dedifferentiation and epithelial–mesenchymal transition serve as sequential enabling transitions for subsequent lineage reprogramming, rather than constituting endpoint metastatic signatures, Xiao et al integrate these phenomena into a unified mechanistic framework. The prognostic superiority of the newly developed DNAmDev (DNA methylation deviation) index over the established mDNAsi stemness score across 9837 TCGA tumor samples underscores this distinction: trans-differentiation and fetal-like plasticity are mechanistically and clinically separable, with the former representing the more direct predictor of metastatic potential and patient survival. The DNAmDev index is derived by non-negative least squares deconvolution of tumor DNA methylation profiles against a reference panel of normal tissue methylation signatures, yielding a quantitative measure of alternative-lineage epigenomic acquisition. Multivariate Cox regression analyses revealed that this index independently predicted the progression-free interval and overall survival, outperforming clinical staging across multiple tumor types, including bladder cancer, lung adenocarcinoma, kidney clear cell carcinoma, prostate adenocarcinoma, and low-grade glioma.

The identification of oncogenic MAPK signaling as the primary genetic driver of lineage trans-differentiation adds substantial translational weight to these findings. Gain-of-function MAPK alterations, germline epidermal growth factor receptor (EGFR) mutations, extrachromosomal oncogene amplification, and kinase fusions are specifically and strongly associated with elevated DNAmDev indices, whereas tumor suppressor loss is not. Mechanistically, MAPK activation induces genome-wide *de novo* DNA methylation at transcription factor binding sites, suppressing original lineage programs while simultaneously licensing ectopic transcription factor activity through permissive chromatin remodeling. Pharmacological anaplastic lymphoma kinase (ALK) inhibition in echinoderm microtubule-associated protein-like 4 (EML4)–ALK^+^ cancer cells reverses trans-differentiation at both transcriptomic and methylomic levels; this reversibility is corroborated *in vivo* through paired pre- and post-tyrosine kinase inhibitor (TKI) biopsy analyses, wherein the TKI response is associated with increased lineage fidelity, while acquired resistance coincides with its loss. These observations provide a compelling molecular rationale for the established clinical benefit of adjuvant TKI therapy in preventing metastasis, as demonstrated in the ADAURA, ALINA, and COMBI-AD trials, and suggest that combination strategies targeting both MAPK oncoproteins and downstream alternative-lineage transcription factors may further improve clinical outcomes. The functional requirement of trans-differentiation for metastatic invasion is additionally supported by siRNA knockdown experiments demonstrating that silencing alternative-lineage master regulators, such as GATA1, GATA3, ETV6, SOX2, and SOX17, significantly impairs the migratory and invasive capacity of human lung cancer cell lines, confirming that trans-differentiation is a functional driver, not merely a correlate, of metastatic progression.

These findings have meaningful implications for precision oncology. The DNAmDev index, which is demonstrable in bulk tumor tissue and potentially adaptable to circulating cell-free DNA platforms, represents a promising prognostic biomarker that may complement existing mutation- and methylation-based liquid biopsy approaches.^5^ Its integration with cell-free DNA methylation-based minimal residual disease (MRD) detection frameworks, a methodology previously developed and validated by the same group in colorectal cancer,^5^ may further increase sensitivity for the early detection of metastatic relapse and the stratification of patients who would benefit from adjuvant systemic therapy. Nevertheless, important limitations define a clear agenda for future investigations. Direct *in vivo* manipulation experiments, such as lineage tracing combined with controlled MAPK activation and inactivation in isogenic models, are needed to formally establish MAPK signaling as sufficient, not merely necessary, for driving trans-differentiation. Whether trans-differentiation facilitates organ-specific metastatic tropism, immune evasion, or adaptation to distal tissue microenvironments remains to be directly tested. The relationship between lineage trans-differentiation and clinically observed histological transformation, such as that of EGFR-mutant lung adenocarcinoma evolving toward small cell neuroendocrine carcinoma under therapeutic pressure, presents a particularly compelling and immediately clinically relevant mechanistic question.

Taken together, the work of Xiao et al established a new epigenomic paradigm for cancer metastasis, centered on the concept that progressive lineage infidelity through epigenomically-driven trans-differentiation is the principal mechanism generating metastasis-competent clones. By developing robust, openly available computational tools, such as scLineageFidelity and DNAmDev, and by validating trans-differentiation as a pan-cancer feature across mouse models, human cohort data, organoid systems, and xenograft models, this study fills a critical theoretical void in cancer biology. These findings not only deepen our mechanistic understanding of cancer evolution but also offer concrete, near-term opportunities for improving prognostic stratification and for developing novel therapeutic strategies that target epigenomic transitions, enabling metastatic dissemination in the era of precision oncology.

## CRediT authorship contribution statement

**Cong Zou:** Investigation, Methodology, Writing – original draft. **Caixia Xia:** Methodology, Writing – review & editing. **Hongwei Peng:** Data curation, Methodology, Resources. **Shenjuan Li:** Data curation, Investigation, Validation. **Jia Shang:** Formal analysis, Methodology. **Wan Xiang:** Data curation, Investigation, Validation. **Haoli Ma:** Software, Visualization. **Fangjin Chen:** Data curation, Software, Visualization. **Xinyue Cao:** Conceptualization, Funding acquisition, Project administration, Supervision, Writing – original draft. **Gang Wang:** Conceptualization, Funding acquisition, Project administration, Supervision, Writing – review & editing.

## Funding

This study was supported by the 10.13039/501100001809National Natural Science Foundation of China (No. 82573858) and the Research Fund of Zhongnan Hospital of Wuhan University (Hubei, China) (No. CXPY202555). The funders played no role in the study design, data collection and analysis, decision to publish, or preparation of the manuscript.

## Conflict of interests

The authors declared no conflict of interests.
